# Rapid, label‐free enrichment of lymphocytes in a closed system using a flow‐through microfluidic device

**DOI:** 10.1002/btm2.10602

**Published:** 2023-09-25

**Authors:** Anton Mukhamedshin, Riley C. Reddington, Mai T. P. Dinh, Kumar Abhishek, Mubasher Iqbal, Marc Manheim, Sean C. Gifford, Sergey S. Shevkoplyas

**Affiliations:** ^1^ Department of Biomedical Engineering University of Houston Houston Texas USA; ^2^ Halcyon Biomedical, Incorporated Friendswood Texas USA

**Keywords:** cell separation, cellular therapy, leukapheresis, lymphocytes, microfluidics

## Abstract

The majority of adoptive cellular therapies are produced from peripheral mononuclear cells obtained via leukapheresis and further enriched for the cells of interest (e.g., T cells). Here, we present a first‐of‐its‐kind closed system, which effectively removes ~85% of monocytes and ~88% of platelets, while recovering ~88% of concentrated T cells in a separate output stream, as the leukapheresis sample flows through a microfluidic device at 5 mL/min. The system is driven by a common peristaltic pump, enabled by a novel pressure wave dampener, and operates in a closed bag‐to‐bag configuration, without requiring any specialized, dedicated equipment. When compared to standard density gradient centrifugation on paired samples, the new system demonstrated a 1.5‐fold increase in T cell recovery and a 2‐fold reduction in inter‐sample variability for this separation outcome. The T cell‐to‐monocyte ratio of the leukapheresis sample was increased to 20:1, whereas with density gradient processing it decreased to 2:1. As a result of superior purity and/or gentler processing, T cells enriched by the system showed a 2.7‐times higher fold expansion during subsequent culture, and an overall 3.5‐times higher cumulative yield. This centrifugation‐free and label‐free closed system for enriching lymphocytes could significantly simplify and standardize the manufacturing of life‐saving cellular therapies.

**Translational Impact Statement**

The technology innovations described herein are highly significant because they enable a truly closed disposable system for enriching lymphocytes that does not require magnetic or fluorescent cell labeling, centrifugation‐based cell processors, nor any other complex dedicated equipment. This new system has the potential to transform cell therapy manufacturing and other applications where cell isolation is crucial. Taken more broadly, this manuscript demonstrates how microfluidic technologies can be applied in clinically‐relevant applications to outperform traditional methods and improve outcomes for researchers and, ultimately, patients.

## 1 INTRODUCTION

Adoptive cellular therapies have emerged as breakthrough treatments for a host of devastating diseases affecting millions of patients worldwide.^1^, ^2^ Lymphocyte subsets in particular—for example, CD3^+^ T cells,^3^, ^4^ CD4^+^ regulatory T cells,^5^, ^6^ or CD56^+^ NK cells^7^, ^8^—are now used to manufacture a rapidly increasing number of potentially curative treatments against cancer, autoimmune diseases, and other disorders. Particularly exciting are the chimeric antigen receptor (CAR) T cell therapies, which have shown unprecedented efficacy in treating hematologic and solid malignancies.^1^, ^9^, ^10^ These therapies utilize the patient's own T cells, enhanced by genetic modification and expanded ex vivo, to destroy cancerous cells upon reinfusion.^2^

The initial step in manufacturing any T cell‐based therapy is the collection of lymphocytes from the patient, typically performed via leukapheresis. During this procedure, the patient's blood is continuously passed through a centrifugation‐based apheresis machine to separate white blood cells (WBCs)—preferentially mononuclear cells (MNCs), that is, lymphocytes and monocytes—from the rest of the blood components which are selectively returned to the patient.^10^, ^11^ The cell composition of leukapheresis units varies greatly, depending on patient/donor characteristics, type of the apheresis machine used and collection parameters.^12^ However, one should typically expect a concentrated suspension of several billion WBCs comprising mostly lymphocytes (65%–85%), but also a significant fraction of monocytes (15%–35%), some granulocytes (<10%), and a substantial number of residual red blood cells (RBCs; usually <5% hematocrit) and platelets (PLTs; often >10^6^/μL).^13^, ^14^, ^15^ The presence of contaminating cells represents a significant problem for downstream manufacturing steps, and decreasing the prevalence of monocytes and PLTs has been shown to improve subsequent activation and growth of T cells in culture,^15^, ^16^, ^17^ as well as to reduce the number of viral particles required for the crucial transduction step used in CAR‐T therapies.^15^ Therefore, leukapheresis units routinely undergo further purification to deplete these unwanted contaminants and increase the purity of the T cells in the final product.^18^, ^19^

Separation methods utilizing natural differences in cell density and size (such as density gradient centrifugation^20^ or elutriation^14^) do not rely on cell labeling and therefore are often simpler, faster, and less expensive than antibody‐based approaches using magnetic or fluorescent cell sorting.^21^, ^22^ However, the yield and purity of these methods are often comparatively low because they lack the resolution required to fully differentiate lymphocytes from other blood cells—which may also overlap to some degree, in density in particular. Additionally, these methods rely on centrifugation, which damages cells of all types^23^, ^24^, ^25^, ^26^, ^27^ and introduces a high degree of variability from sample to sample. Automated closed systems for T cell purification based on these centrifugation‐based methods are available but require relatively expensive machinery that is not commonly available to most researchers, while manual approaches can be laborious and significantly increase the risk of microbial contamination as well as human error.^28^

A considerable amount of research effort has been aimed at overcoming the above limitations using a variety of microfluidic size‐based separation methods, with the elusive goal of achieving sufficient capacity to process a clinically‐relevant volume of blood sample in a reasonable amount of time.^29^, ^30^, ^31^, ^32^ We have ourselves previously developed a high‐throughput microfluidic device based on controlled incremental filtration (CIF)^33^, ^34^ that could remove >70% of RBCs and PLTs while recovering ~85% of the T cells from leukapheresis samples driven through the device by simple gravity.^13^ However, none of these previously‐published microfluidic approaches could also remove the culture‐contaminating monocytes, nor process the sample in a truly closed (non‐syringe‐based) system configuration when higher flowrates are desired.

Here, we describe the design and validation of a high‐throughput CIF‐based microfluidic device, capable of bag‐to‐bag closed system purification and concentration of lymphocytes from minimally diluted leukapheresis units. The flow of leukapheresis sample through the device was driven by a peristaltic pump at a rate of 5 mL/min. The device removed ~85% of monocytes and ~88% of PLTs, while recovering ~88% of T cells and concentrating them approximately 4‐fold. The levels of both monocyte depletion and T cell recovery for the device were significantly higher than that for the standard density gradient centrifugation method, which was performed on paired samples. Importantly, T cells enriched by the CIF device expanded in culture significantly faster (~2.7 times higher fold‐expansion) compared to samples processed via density gradient centrifugation, and—when also taking into account the improved initial separation efficiency—generated far more total cells overall (~3.5 times higher cumulative yield) at the end of culture.

## 2 MATERIALS AND METHODS

### 2.1 Leukapheresis samples

Leukapheresis units (*n* = 6) were collected from healthy, consenting volunteers by commercial vendors using a Spectra Optia® Apheresis System (Terumo BCT, Lakewood, CO) with the Continuous Mononuclear Cell Collection protocol. Two units purchased from AllCells (Alameda, CA) and a one from HemaCare (Northridge, CA) were shipped overnight in an insulated box at room temperature (15–20°C). An additional three units from AllCells were picked up locally and processed on the day of collection. All leukapheresis samples were passed through a 40 μm aggregate filter (SQ40; Haemonetics Corp, Boston, MA) prior to processing. Sterile phosphate‐buffered saline (PBS) with 0.8% (w/v) poloxamer Kolliphor® P 188 (Sigma‐Aldrich, St. Louis, MO) was used to dilute the leukapheresis samples such that ${\mathrm{WBC}}_{\text{adjusted}}={\mathrm{WBC}}_{\text{count}}-0.9\times {\text{LYMPH}}_{\text{count}}$ was equal to ~8 × 10^6^ cells/mL. The resulting dilution ratios ranged from 1:1 to 1:2.6 (sample: diluent).

### 2.2 Microfluidic device fabrication

As described previously,^13^, ^34^ the design of the ‘microchannel layer’ of the CIF device was generated in MATLAB (The MathWorks Inc, Natick, MA) and transferred using photolithography into a 140 μm‐thick layer of photoresist (SU‐8 3050; Kayaku Advanced Materials Inc, Westborough, MA) that was spun on a 4‐inch silicon wafer (University Wafer, South Boston, MA). This wafer served as the master mold to produce inverse replicas in poly(dimethylsiloxane) (PDMS, 8:1 base:crosslinker; Sylgard 184, Dow Corning Corp, Midland, MI). A second master wafer containing a system of larger channels for collecting and distributing the filtrate and retentate outputs was replicated in PDMS (10:1 base:crosslinker) to create a ‘manifold layer.’ Inlet and outlet ports were created using biopsy punches of appropriate size (Robbins Instruments, Houston, TX). To assemble a CIF device, the microchannel layer was bonded to a PDMS‐coated Petri dish (flat substrate), and then the manifold layer was bonded atop the microchannel layer, using dry air plasma (PDC‐001, Harrick Plasma, Ithaca, NY). An inverted 30 mL plastic bottle (Wheaton® Leak Resistant Bottle, DWK Life Sciences, Millville, NJ) equipped with a Luer‐lock bottle cap (JGC‐512‐10, Jensen Global Inc, Santa Barbara, CA) was attached at the input port of the CIF device, via a 3‐way Luer‐lock connector, to serve as a pressure wave dampener. The assembled CIF device was incubated with a 1% (w/v) aqueous solution of mPEG‐silane (MW 5000; Laysan Bio Inc, Arab, AL) for 30 min, and then with 1% (w/v) human serum albumin (HSA; Sigma‐Aldrich) solution in isotonic PBS overnight.

### 2.3 Microfluidic device operation

The inlet of the CIF device was connected to the input blood bag (Fenwal, Inc., Lake Zurich, IL) with a two‐foot length of silicone peristaltic tubing (size 14, Masterflex™, Cole‐Parmer, Vernon Hills, IL). A peristaltic pump with a 3‐roller head (BT100S/YZ15; Golander, Duluth, GA) was used to drive the sample from the input bag through the CIF device. The outlets of the CIF device were connected via polyurethane tubing (Scientific Commodities, Lake Havasu City, AR) to three separate collection bags (PEGA®, Venner Medical (Deutschland) GmbH, Danischenhagen, Germany). After priming the device and dampener with PBS (i.e. pumping PBS through the device for ~3 min until the fluid level in the dampener reached a steady‐state), the device outlets were clamped, and the input bag was filled with 20 mL of leukapheresis sample and the required amount of diluent (see above). The device outlets were then unclamped, and the sample was pumped through the device at 5 mL/min. Once the final ~0.25 mL of the input sample had reached the end of the peristaltic‐pump tubing, the pump was turned off, allowing the pressurized buffer in the dampener to wash through the device and flush residual cells remaining within the ‘dead volume’ of the device into their respective collection bags.

### 2.4 Density gradient centrifugation

Density gradient centrifugation was performed according to the manufacturer's instructions (Lymphoprep™, StemCell Technologies, Vancouver, Canada). For each procedure, 20 mL of the leukapheresis sample was diluted 1:1 with PBS and split equally between two 50 mL conical tubes, each containing 15 mL of Lymphoprep™ medium. The tubes were centrifuged at 800 × g for 30 min with the brake off. The plasma layers (top) and RBC layers (bottom) were discarded, and the middle layers containing enriched MNCs were transferred to a new tube and washed thrice with PBS (300 × g for 10 min, brake low). The final pellet was re‐suspended in PBS to yield a total volume of 5 mL.

### 2.5 Cell counting

For all samples, a complete blood count with five‐part differential was performed using a hematology analyzer (XS‐1000i, Sysmex America, Inc., Mundelein, IL). Additionally, WBC and RBC counts were assessed via direct visual counting of cells in microscopic images of the sample in a hemacytometer (C‐CHIP, Incyto Co., Covington, GA).

### 2.6 Flow cytometry analysis

RBCs were lysed using appropriate buffer (BD Pharm Lyse™, #555899, BD Biosciences, Franklin Lakes, NJ). Each sample was adjusted to a concentration of 10^7^ WBCs/mL with cold (4°C) FACS buffer (PBS with 2% BSA), and a 100 μL aliquot was placed into a polystyrene tube. Aliquots of stock antibody solutions (20 μL) of anti‐CD45^+^ APC/Cyanine7, anti‐CD3^+^ FITC, anti‐CD4^+^ PE/Cyanine7, anti‐CD8^+^ APC, anti‐CD197(CCR7)^+^ APC/Cyanine7 (BioLegend, San Diego, CA, USA), and anti‐CD14^+^ PE, anti‐CD69^+^ PE, anti‐CD45RA^+^ PE, anti‐CD25^+^ APC‐Cy™7 (BD Biosciences), were added to each sample, and samples were incubated at 4°C for 30 min in the dark. Next, samples were washed (twice) with 2 mL of FACS buffer by centrifugation at 300 × g for 5 min at 4°C. The supernatant was discarded, and labeled cells were re‐suspended in 100 μL of FACS buffer. A 5 μL aliquot of the viability dye (7AAD, BioLegend) was added to each sample and incubated for 10 min at room temperature in the dark. Samples were analyzed using a flow cytometer (Novocyte 2006R, Ace Bioscience, San Diego, CA, USA) with all necessary controls (unstained and compensation).

### 2.7 Expansion of T cells in culture

Isolated T cells were suspended at 10^6^ cells/mL in RPMI 1640 media with L‐glutamine (Caisson Labs, Smithfield, UT, USA) supplemented with 10% fetal bovine serum (FBS; Sigma‐Aldrich), 1% (v/v) of penicillin/streptomycin solution, 2.5 μg/mL amphotericin B (Caisson Labs), 25 mM HEPES (Caisson Labs), 100 ng/mL Interleukin (IL)‐2 (PeproTech, Cranbury, NJ), and 25 μL/mL ImmunoCult™ CD3/CD28 T cell activator (StemCell Technologies). Cells were seeded (10^6^ cells/well) in a non‐treated, flat‐bottom, 24‐well tissue culture plate (CellTreat, Pepperell, MA, USA) and incubated at 37°C with 5% CO_2_ for 3 days. On day 3, cells were collected from the plate, spun down by centrifugation at 250 × g for 10 min, re‐suspended in culture media without activator (0.25 × 10^6^ cells/mL), and seeded in a new 24‐well plate (0.25 × 10^6^ cells/well). Culture media supplemented with 100 ng/mL of IL‐2 was replaced every 3 days. When the total cell count for the sample reached 10^7^, the cells were re‐suspended at a concentration of 10^6^ cells/mL and culture continued in a 250 mL suspension culture flask (CellTreat, Pepperell, MA, USA). Total numbers of viable cells were determined using an automated cell counter with Trypan Blue (Countess II, ThermoFisher, Waltham, MA) before each media exchange.

### 2.8 Computational fluidic dynamics simulations

Finite element analysis simulations of fluid flow in filtration gaps of the CIF devices were performed using COMSOL Multiphysics (COMSOL, Inc., Burlington, MA). Simulations were carried out in 2D using actual geometry of the device, assuming a laminar stationary flow, a Newtonian fluid, and no‐slip boundary conditions.

### 2.9 Statistical analysis

All values are expressed as mean ± standard deviation. Statistical significance (defined as *p* < 0.05) of the observed differences was determined using a paired *t*‐test.

## 3 RESULTS

### 3.1 Design and operation of the microfluidic device for label‐free enrichment of lymphocytes

**FIGURE 1:**
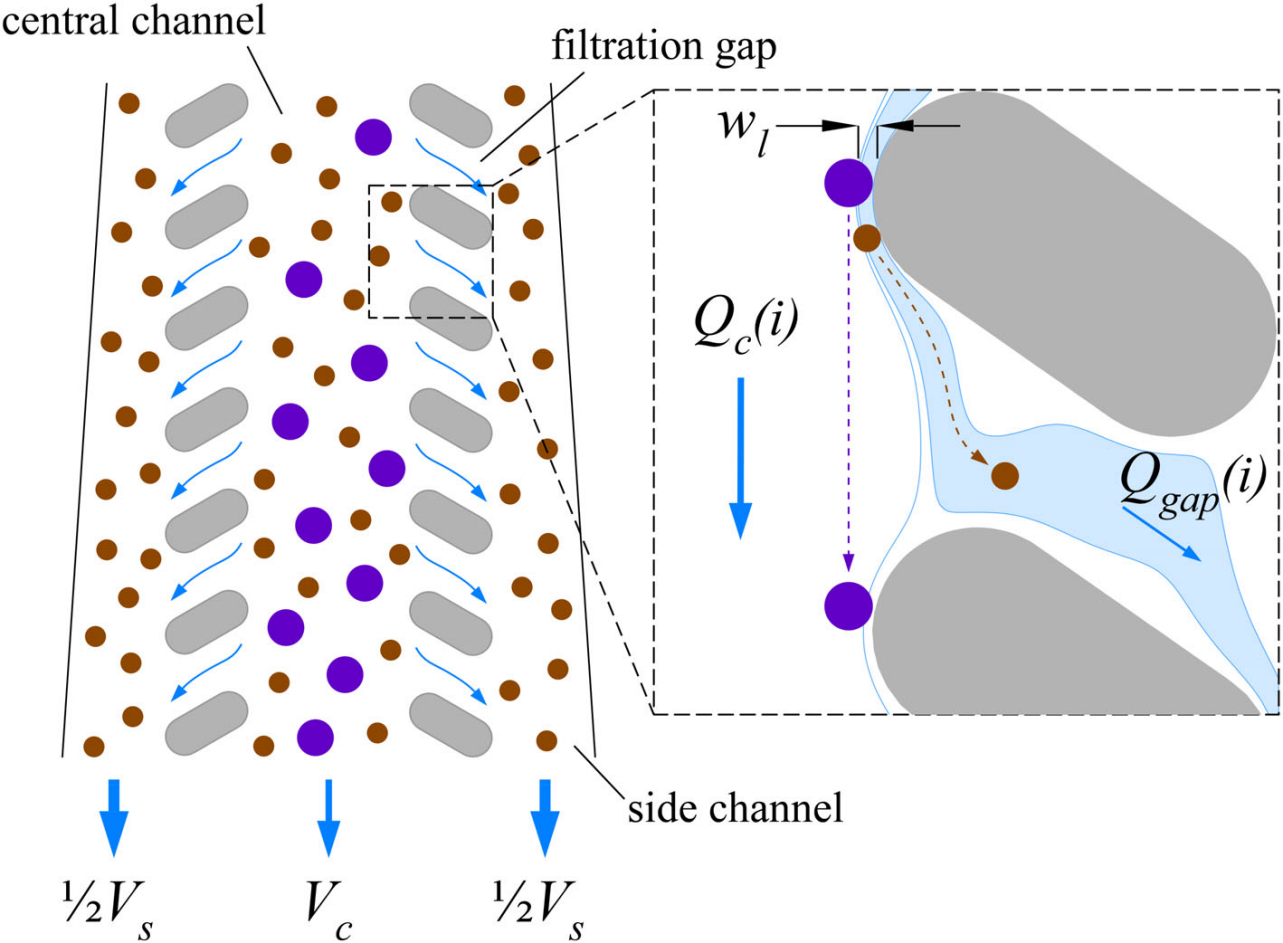
Schematic illustration of the CIF separation principle. A small fraction of the central channel flow is siphoned through each gap along the length of a CIF element. Particles with an effective size sufficiently large to not be pulled into the extracted fluid lamina remain in the central channel. Smaller particles follow the fluid lamina into the side channels.

A typical CIF element comprises a central (retentate) channel that is separated from two adjacent side (filtrate) channels by a series of filtration gaps on either side (Figure 1). At each filtration gap i, a small fraction $f_{\mathrm{gap}}\left(i\right)$ of the central channel axial flow $Q_{c}\left(i\right)$ is siphoned into the side channel ($Q_{\mathrm{gap}}\left(i\right)=f_{\mathrm{gap}}\left(i\right)Q_{c}\left(i\right)$) due to the gradual, precisely calculated changes in channel dimensions (Figure 1, insert). The mathematical framework for these calculations has been described previously in detail,^33^, ^34^ and they serve to maintain a constant width of the flow lamina ($w_{l}$) siphoned from the central channel through each filtration gap of the CIF element, regardless of the progressive changes in central and side channel widths which are required to produce this siphoning. A constant width of the extracted flow lamina ensures a uniform size threshold for the cells that are pulled into the side channels through each gap (Figure 1, insert), thereby ensuring consistent concentration/filtration performance throughout the entire length of a given CIF element.

The fluid dynamics within the filtration gaps are rather complex, even when flowing a Newtonian liquid, and the configuration of the fluid velocity field within a gap varies with the level of siphoning (see Figure S1 for a COMSOL Multiphysics simulation). For $f_{\mathrm{gap}}\left(i\right)=0$ (i.e., a design with no net flow through the filtration gap, $Q_{\mathrm{gap}}\left(i\right)=0$), two symmetrical recirculation zones form within the gap (Figure S1a). With increasing $f_{\mathrm{gap}}\left(i\right)$, the flow through the gap $Q_{\mathrm{gap}}\left(i\right)$ also increases, and consequently, the recirculation zones gradually disappear (Figure S1b–d). Some cells may become entrapped in the recirculation zones, which renders these zones visible (e.g., refer to Video S6). However, in a CIF design there is always a continuous, non‐zero flow through each gap ($Q_{\mathrm{gap}}\left(i\right)>0$), transporting a net flux of fluid—along with sufficiently small cells—from the central channel to the side channels (Figure 1, insert).

Notwithstanding cell crowding effects, the maximum fraction of cells smaller than the size threshold that are removed with the filtrate fluid is equal to $\mathrm{FR}/\left(1+\mathrm{FR}\right)$, where FR is the ‘flow ratio’ of the CIF element defined as the cumulative output volume of filtrate ($V_{S}$) divided by the volume of retentate ($V_{C}$). (For example, for a CIF element with a FR=10, this theoretical maximum removal would be ~91%.)

**FIGURE 2:**
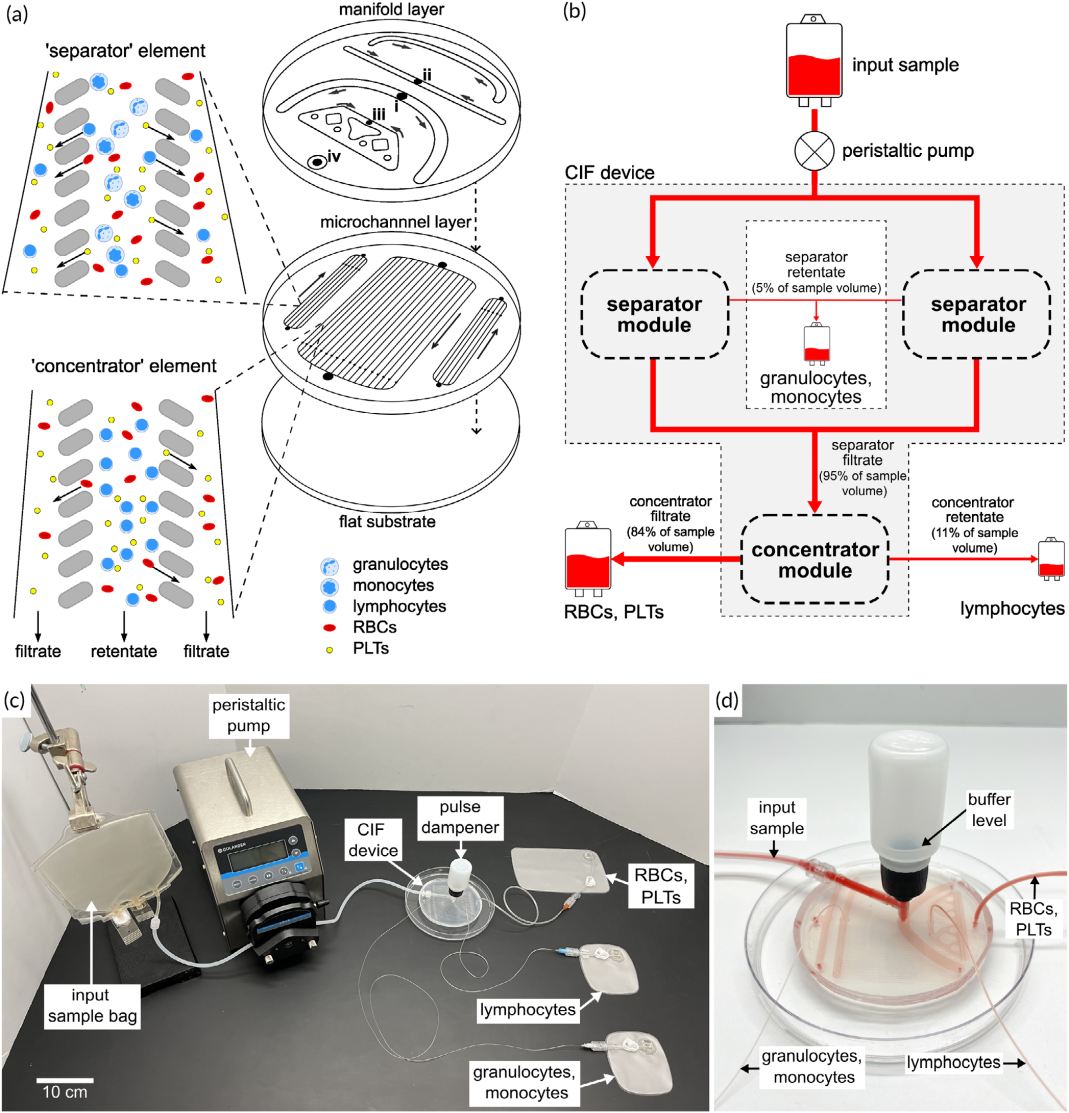
Design and operation of the CIF device. (a) An assembled CIF device consisted of three PDMS layers sealed on top of one another (right). The ‘microchannel layer’ included two ‘separator’ modules each comprising 10 CIF elements (top left inset) that selectively depleted monocytes and granulocytes (retentate), while recovering lymphocytes, RBCs, and PLTs (filtrate), and a ‘concentrator’ module comprising 48 CIF elements (bottom left inset) that filtered out RBCs and PLTs (filtrate) and recovered lymphocytes (retentate). The ‘manifold layer’ channels connect the inlets and outlets of the CIF modules such that the sample entering through the input port (i) was first driven through the twin separator modules. The monocyte‐ and granulocyte‐rich retentate from the separators was driven through exit port (ii), and the filtrate was directed to the concentrator module. The lymphocyte‐rich retentate of the concentrator module was collected via exit port (iii), and the filtered‐out RBCs and PLTs exit the device through port (iv). (b) Schematic illustration of the CIF device workflow: the separator modules (~20:1 flow ratio) separated the input sample into monocyte/granulocyte‐rich retentate and smaller cell‐containing filtrate that subsequently traversed the concentrator module (~8:1 flow ratio), in which lymphocytes (and any other residual WBCs) were concentrated into a product ~11% of the total volume processed. (c) Photograph of the CIF‐based closed system for bag‐to‐bag processing of leukapheresis samples driven by a peristaltic pump. (d) Photograph of an assembled CIF device with a 30 mL pulse dampener. The dampener is pressurized with PBS buffer prior to processing the blood sample and serves to attenuate the pressure oscillations generated by the peristaltic pump—thereby maintaining stable fluidic streamlines within the flow channels of the device.

The CIF‐based microfluidic device designed for this study purified lymphocytes in two stages by: first selectively removing larger WBCs (granulocytes, monocytes) from the input stream of the leukapheresis sample, and then concentrating the remaining lymphocytes while filtering out RBCs and PLTs. To increase the throughput at a given driving pressure, multiple CIF elements with the same size threshold were combined in parallel to form modules. The multiplexed device consisted of three distinct modules: two identical ‘separator’ modules connected in parallel, and a downstream ‘concentrator’ module connected in series (Figure 2a,b). Each separator module was comprised of 10 individual CIF elements (a total of 20 separator elements per device) with a size threshold chosen to let the vast majority of lymphocytes, RBCs, and PLTs escape in the filtrate while concentrating monocytes and granulocytes in the retentate. These separator elements had a FR=20, and, therefore, could theoretically initially recover up to ~95% of the lymphocytes present in the original leukapheresis sample. The concentrator module consisted of 48 individual CIF elements designed to retain nearly all of the now highly enriched lymphocytes in its retentate output stream, while letting RBCs and PLTs escape into the filtrate. The concentrator elements had a FR=8, and, therefore, could theoretically remove ~89% of the RBCs and PLTs from the lymphocyte‐rich sample produced by the separators (note a small portion of RBCs and PLTs are also lost to the separator retentate).

During each experiment, the flow of leukapheresis sample through the CIF device was driven by a peristaltic pump (Figure 2b,c; see also Video S1). To compensate for the pressure oscillations inevitably generated by such a pump, the CIF device included an integrated pressure pulse dampener (Figure 2d). The size of the dampener (30 mL) was selected to serve as an effective shock absorber for the magnitude of the cyclical pressure waves generated by the specific peristaltic pump/head/tubing combination that were used in this study (see Figure S2 and Videos [Link], [Link]), and to trap (during priming of the device) an amount of buffer suitable to wash out the residual cells remaining in the device at the end of sample processing. Due to the use of a peristaltic pump, the CIF device could process the leukapheresis sample bag‐to‐bag, under sterile (closed system) conditions (Figure 2c).

The system demonstrated consistent separation performance for flow rates up to ~5 mL/min, which corresponded to a driving pressure of ~4.7 psi. At ~6 mL/min (~5.6 psi) the negative effects of pressure‐induced deformation began affecting the separation performance of the prototype CIF devices which were fabricated from a soft elastomer (PDMS). Complete operational failure was only observed at the point of device delamination, which typically occurred at flow rates ranging 12–15 mL/min (~11–14 psi), depending on the quality of plasma bonding of each specific device prototype. Given the layout of the CIF elements comprising the device, the largest Reynold's number experienced within the device occurred within the initial length of the ‘separator’ elements and the lowest was at the exit of the ‘concentrator’ elements (please see Figure 2a,b for a schematic of the device architecture). By applying the hydraulic diameter‐based approach to a CIF device operating at a flow rate of 5 mL/min,^35^ the Reynold's number can be estimated as ~22.2 at the entry (234 μm × 140 μm cross‐section) and ~1.98 at the exit (70 μm × 140 μm) of the ‘separator’ elements, and ~12.6 at the entry (120 μm × 140 μm) and ~1.81 at the exit (70 μm × 140 μm) of the ‘concentrator’ elements.

### 3.2 Comparing the cell separation efficiency of the CIF device with that of standard density gradient centrifugation

The T cell enrichment performance of the CIF device was compared to that of the standard density gradient centrifugation method on paired samples, in a split‐unit study using six leukapheresis units, three of which were shipped overnight while three were processed on the day of collection. The products produced by the CIF device had on average a ~4‐times higher concentration of T cells (111.2 ± 34.7 × 10^6^/mL) than in the initial leukapheresis units (27.9 ± 5.6 × 10^6^/mL), without the need for pelleting the sample as is done with density gradient centrifugation.

The CIF device demonstrated a markedly higher recovery for lymphocytes overall (by 24 absolute percentage points) and T cells specifically (by 28 percentage points, or 1.5‐times) than density gradient centrifugation, and these differences were statistically significant (Table 1). Moreover, the recoveries for the CIF device were substantially higher than for the density gradient for each of the six leukapheresis units individually (see Table S1), and the standard deviation in recoveries measured for the CIF device (4%–5%) was much smaller than for the density gradient method (10%–16%). The CIF device was also significantly more effective than the density gradient at depleting monocytes (by 60 percentage points, or more than three times). Both methods demonstrated a similar level of PLT removal, and the density gradient was significantly better at depleting RBCs (Table 1). Because of the way they are collected, leukapheresis samples typically contain few granulocytes. Both separation methods appeared to deplete this already small subpopulation, but the concentration of residual granulocytes in the products was too low to calculate removal percentages accurately.

**TABLE 1:** Cell separation performance of the CIF device in comparison to standard density gradient centrifugation.

	Density gradient centrifugation	CIF device
Recovery		
Lymphocytes	61 ± 10%^a^	85 ± 5%^a^
T cells	60 ± 12%^a^	88 ± 5%^a^
CD4^+^ T cells	65 ± 16%	85 ± 4%
CD8^+^ T cells	62 ± 11%^a^	84 ± 4%^a^
Removal		
Monocytes	25 ± 15%^a^	85 ± 5%^a^
PLT	83 ± 10%	88 ± 3%
RBC	96 ± 6%^a^	77 ± 19%^a^

*Note*: Values shown are mean ± standard deviation (*n* = 4 for CD4^+^ and CD8^+^, *n* = 6 for all other values).

^a^ Statistically significant difference between the two methods.

**FIGURE 3:**
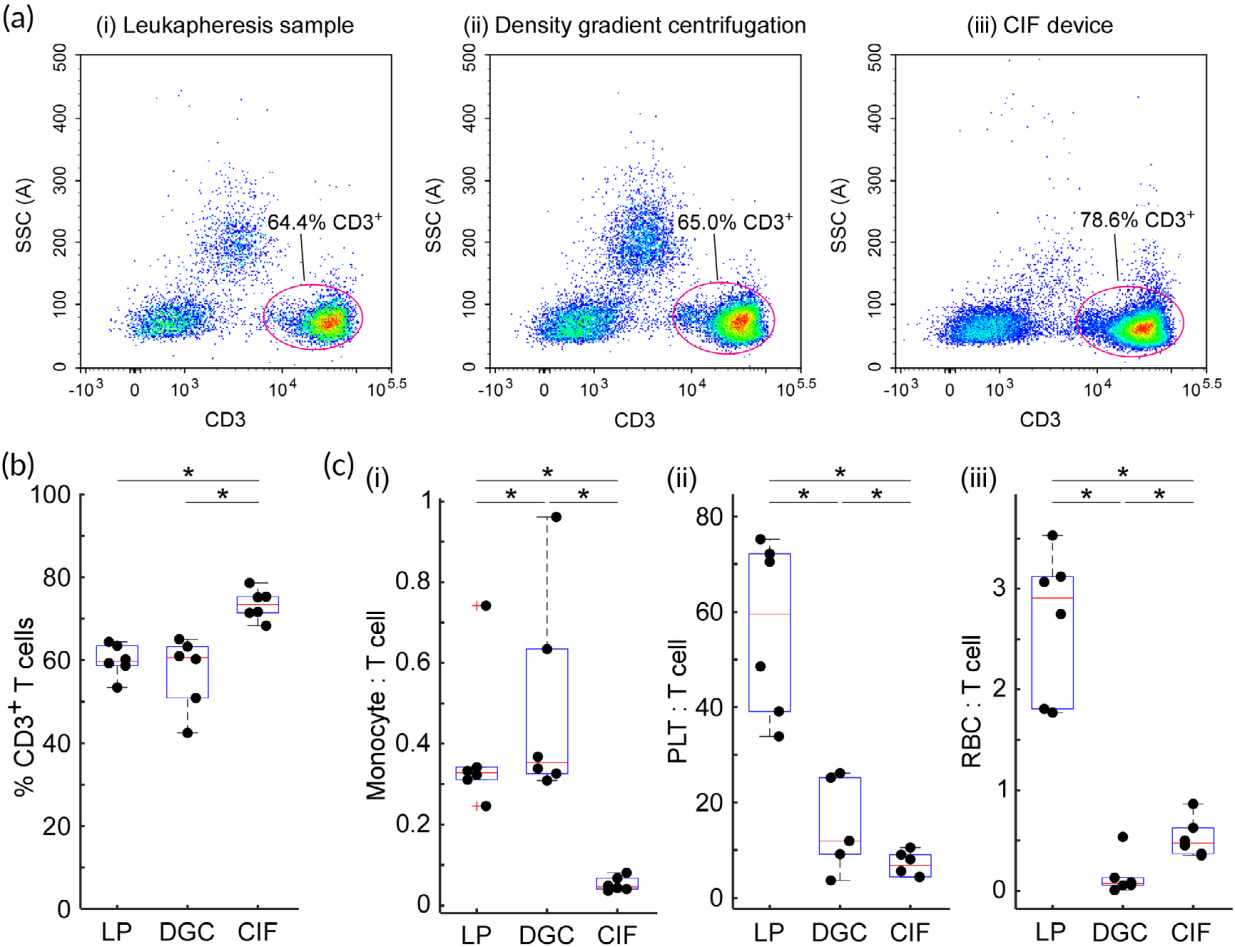
Comparison of T cell enrichment for the CIF device versus the density gradient centrifugation (DGC) method. (a) Representative scatterplots showing the percentage of live CD45^+^ cells that were CD3^+^ in the initial leukapheresis (LP) sample and after separation using DGC or the CIF device. (b) Boxplots showing the purity of CD3^+^ T cells and (c) the number of contaminating cells per each T cell in the sample for (i) monocytes, (ii) PLTs, and (iii) RBCs before and after processing. Values are shown as mean ± standard deviation (*n* = 6); *statistically significant difference between the samples.

Figure 3 illustrates the effect of cell separation method on the purity of isolated T cells. Overall cell viability was slightly higher for the CIF product (96.9 ± 3.9%) than for either the initial leukapheresis sample (96.0 ± 5.7%) or the density gradient product (96.1 ± 5.0%), but the differences were not significant. As a percent of live WBCs, the fraction of T cells was significantly higher in the CIF product (73.4 ± 3.3%) than either the initial leukapheresis sample (59.9 ± 3.6%) or in the density gradient product (57.1 ± 8.0%) (Figure 3a,b).

Both separation methods significantly altered the level of contaminating blood cells (monocytes, PLTs, and RBCs) that are known to affect the downstream steps of a typical cellular therapy manufacturing process (Figure 3c).^15^, ^16^ The CIF product contained significantly fewer (~7‐times) monocytes per T cell (0.05 ± 0.02) than were in the initial leukapheresis sample (0.38 ± 0.16). In contrast, the density gradient method significantly *increased* the number of monocytes per T cell (to 0.49 ± 0.24), and the ~9‐times difference in the monocyte/T cell ratio between the two methods was significant. The CIF device was also significantly more effective in lowering the number of PLTs per T cell from 56.5 ± 16.7 in the initial leukapheresis sample to only 7.0 ± 2.4 (versus 14.6 ± 8.3 for the density gradient method). Although it was able to significantly reduce the number of RBCs per T cell (from 2.67 ± 0.67 to 0.52 ± 0.18), the CIF device was not as effective as the density gradient method (0.14 ± 0.18), though this result was affected somewhat by one overnight unit that had an anomalously low RBC removal in the CIF arm, likely because many RBCs were no longer discocytes (see Table S1). Other than that single RBC removal outlier, we found no significant differences between the leukapheresis units processed on the day of collection versus shipped overnight.

### 3.3 Effect of cell separation method on the expansion of purified T cells in culture

**FIGURE 4:**
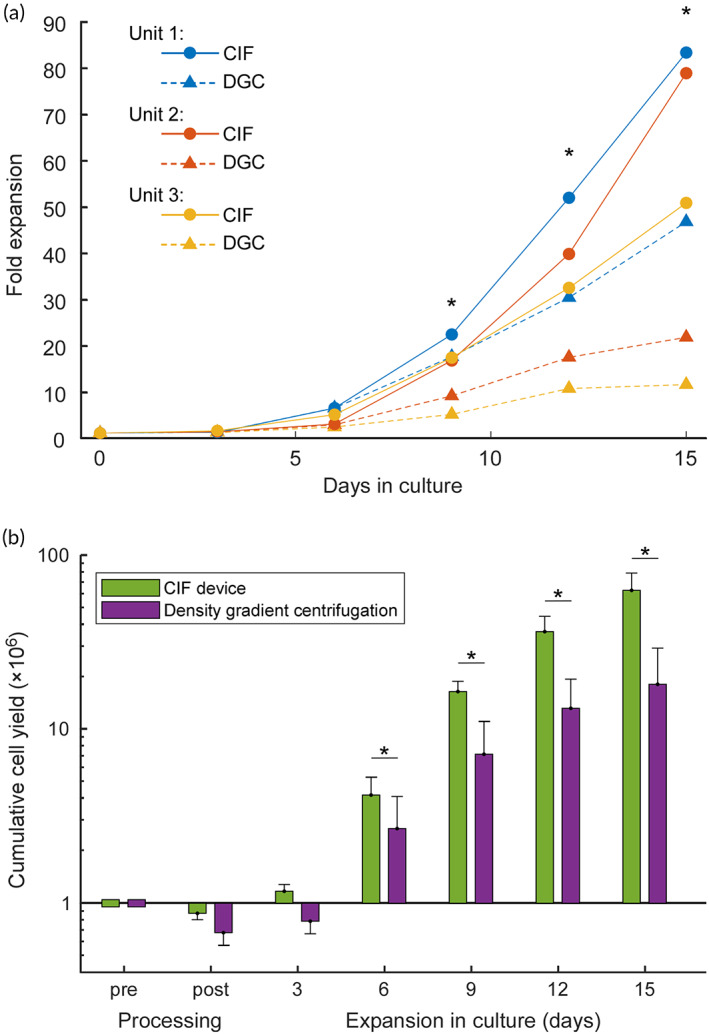
Effect of cell separation method on the ability of T cells to expand in culture. (a) Fold expansion of T cells isolated from leukapheresis samples using the microfluidic device (CIF) or standard density gradient centrifugation (DGC) method. (b) Cumulative cell yield for every 10^6^ T cells initially present in the leukapheresis sample. For each leukapheresis unit that was cultured, the yield was calculated using the experimentally measured T cell recovery post‐processing (see Table S1) and the corresponding fold expansion curve (see panel a). Values shown are mean ± standard deviation (*n* = 3); *statistically significant difference between the CIF device and the density gradient method.

To investigate the effect of the lymphocyte enrichment process on the ability of purified T cells to expand in culture, paired samples produced by the CIF device and density gradient centrifugation from three leukapheresis units were activated with soluble anti‐CD3^+^/anti‐CD28^+^ antibody complexes for 3 days and expanded over 12 additional days in IL‐2 culture. The T cells purified using the CIF device showed somewhat higher activation level (i.e. percentage of CD25^+^CD69^+^ cells) after 3 days of stimulation (37 ± 17% vs. 29 ± 12%), but the difference was not significant. The CIF‐derived cells followed a quasi‐exponential (average *R* ^2^ = 0.9573) growth curve for the entire duration of culture, while the expansion of cells produced by the density gradient method tended to slow down after day 9 (Figure 4a). One leukapheresis unit was sampled and processed twice—once on the day of collection and again after an overnight hold (to simulate shipping). The difference in terms of subsequent fold expansion of those processed cells in culture was negligible (see Table S1). After 15 days, the average fold expansion for CIF‐derived cells was ~2.7‐times higher than for the density gradient method (71 ± 14‐fold vs. 27 ± 15‐fold). For each unit, there were more CIF‐derived cells on day 12 than density gradient‐derived cells on day 15 (Figure 4a).

As expected, at the end of culture, virtually all cells were T cells (CD3^+^) for both methods (99.0 ± 0.5% for CIF and 98.8 ± 1.0% for density gradient). There was no significant difference in the memory immunophenotype (CD45RA^+^ CCR7^+^ naïve, CD45RA^−^CCR7^+^ central memory, CD45RA^−^CCR7^−^ effector memory, and CD45RA^+^ CCR7^−^ terminally differentiated effector memory) of T cells isolated using either method before or after the expansion (see Table S1). Finally, the total yield—that is, a method's separation recovery (Table 1) multiplied by the fold‐expansion of its separated cells in culture (Figure 4a)—calculated for 10^6^ T cells initially present in the leukapheresis sample was cumulatively ~3.5‐times higher for the CIF device (63 ± 16 × 10^6^ T cells) than for the density gradient method (18 ± 11 × 10^6^ T cells) for the three units whose cells were cultured (Figure 4b).

## 4 DISCUSSION

This study represents a significant breakthrough in the field of microfluidic blood cell separation by demonstrating the isolation of lymphocytes from other blood cells (monocytes, granulocytes, as well as RBCs and PLTs) by size, with high efficiency and throughput from a minimally‐diluted sample driven through the device by a peristaltic pump. Our two‐stage CIF device separated lymphocytes from other WBCs in the leukapheresis sample with 85% recovery and >90% purity, on average. Most previously published microfluidic approaches, including our own,^13^ could not separate lymphocytes from other WBCs at all,^32^ and those that could, had significantly lower separation efficiency. For example, a microstructure filtration device developed by Guo et al. could recover ~53% of lymphocytes with a purity of 62%–68% from a sample of undiluted whole blood (but at a flow rate of only ~8 × 10^−5^ mL/min).^36^ An inertial focusing device implemented by Ramachandraiah et al. could recover 47% of lymphocytes with 91% purity while processing blood diluted ~1:9 (sample:diluent buffer) at a flow rate of 1 mL/min, while requiring RBC lysis.^37^ Using a device with a modified ‘deterministic lateral displacement’ (DLD) design, Yamada et al. could remove ~75% of monocytes from a sample of whole blood diluted ~1:20 and flowing at 0.04 mL/min; however, they did not also purify lymphocytes from RBCs and PLTs.^38^ To the best of our knowledge, CIF is the only microfluidic technology that has both the precision necessary to separate lymphocytes from all other types of blood cells by size without requiring cell lysis, along with the relatively large feature sizes to achieve practical flow rates.

The 5 mL/min (300 mL/h) flowrate combined with minimal sample dilution (1:1–1:2.6) demonstrated in our study represents a significantly higher per‐cell processing rate than other microfluidic separation methods, which typically must dilute input blood samples an enormous degree (e.g., ≫10‐fold in the case of inertial focusing) in order to show effective performance.^30^, ^31^, ^32^, ^39^, ^40^, ^41^ Further, this volumetric throughput has been achieved in soft elastomeric prototype devices, with an extremely low driving pressure (~5 psi) relative to what would be required by alternative microfluidic approaches having a similar footprint, as CIF‐based systems do not suffer from the presence of features smaller than 20 μm,^30^, ^31^ and can therefore be readily fabricated with relatively large channel depth (here, ~140 μm).^13^, ^34^, ^42^ The maximum flow rate should be higher still once CIF devices are manufactured from a rigid thermoplastic.

Ultimately, the desired flow rate will strongly depend on the point in the sample processing where a device is implemented. For example, one possible application is to integrate the CIF device directly into the leukapheresis workflow to enrich lymphocytes from the sample as it is collected from the patient/donor. During a typical leukapheresis procedure the sample is accumulated at ~1 mL/min.^43^ The sample dilution required by the CIF device would increase the volume of collected sample by 2‐to‐4‐times for an effective collection flow rate of 2–4 mL/min. Therefore, the 5 mL/min flow rate would allow to enrich lymphocytes at the rate of leukapheresis sample collection, without increasing the procedure duration. If, as demonstrated in this study, a CIF device is used to purify a previously collected leukapheresis sample, a flow rate of 5 mL/min would allow for the processing of a typical 200 mL leukapheresis unit (diluted to 600 mL with buffer) within 2 h. If the CIF device were made of rigid thermoplastic, increasing the driving pressure to just 20 psi would quadruple the flow rate, reducing the processing time to ~30 min.

This study is the first to demonstrate successful operation of a microfluidic cell separation device with flow driven by a peristaltic pump. In our own previous studies, a syringe pump (or a hydrostatic pressure gradient created by gravity, or pressure applied to the input bag pneumatically) was used to drive the sample through the device because of its requirement for stable, oscillation‐free flow.^13^, ^34^, ^42^, ^44^ Similarly, other groups have been utilizing various syringe pump‐based approaches to minimize the pressure/flow oscillations which inevitably degrade the performance of most if not all microfluidic cell separation devices.^32^ However, common syringe pumping methods are not strictly compatible with the closed‐system processing required in the clinical setting.^18^, ^19^ Here we used a simple pulse dampener to minimize the pressure waves generated by the peristaltic pump, to enable sterile and continuous processing of the input sample without any of the complex, high‐pressure, multiple syringe‐based equipment required by other microfluidic systems.^30^, ^31^

Standard separation methods based on density gradient centrifugation, such as Ficoll‐Paque™ PLUS, typically advertise ~60 ± 20% lymphocyte recovery^45^ and in our hands this approach (using another brand of comparable medium^46^) showed a similar recovery for lymphocytes in general (61 ± 10%), and T cells specifically (60 ± 12%). The CIF device outperformed density gradient centrifugation on paired leukapheresis samples significantly (by ~25 absolute percentage points; representing ~40% more lymphocyte cells acquired) recovering 85 ± 5% of lymphocytes and 88 ± 5% of T cells initially present. Additionally, the CIF device displayed much less sample‐to‐sample variability in recovery/removal for most cell types (as evidenced by two to four times smaller standard deviations), suggesting a more consistent and predictable separation performance than density gradient centrifugation.

In addition to a high yield for the cells of interest, cellular therapy manufacturing often requires a substantial depletion of various contaminating cells known to interfere with downstream processing (e.g., PLTs and monocytes for T cell‐based therapies).^15^, ^16^, ^18^ While density gradient processing removes a substantial fraction of PLTs (here, ~83%), the level of removal depends on the number of centrifugation washes performed to remove the mildly toxic density gradient medium from the product.^47^, ^48^ In contrast, the CIF device removed ~88% of PLTs in a single pass, without any centrifugation whatsoever. Importantly, the CIF device also removed most monocytes (85 ± 5%), which, combined with high T cell recovery, resulted in a drastically reduced monocyte:T cell ratio (~0.05). Although the standard density gradient centrifugation method is not designed to separate lymphocytes from monocytes, approximately 25% of monocytes were lost during processing (likely passively, due to the multiple centrifugation steps involved). Combined with the relatively poor recovery of T cells, the monocyte:T cell ratio actually increased from ~0.38 to ~0.49 following density gradient processing. This is a major limitation of the density gradient method as monocytes are well known to interfere with T cell expansion in culture.^15^, ^17^, ^18^ Among conventional, closed‐system technologies, only elutriation can separate lymphocytes and monocytes to some degree, but it is ineffective at separating lymphocytes from contaminating RBCs, and requires highly complex/specialized machinery.^15^ The ability to separate lymphocytes with high yield while removing the vast majority of monocytes and PLTs without centrifugation or magnetic beads, as demonstrated in this study, is a unique feature of our two‐stage CIF device which could be particularly useful in the field of cellular therapy manufacturing.

With ~15 PLTs per T cell (rather than only ~7), and with one monocyte for every two T cells (rather than for every 20), combined with the likely centrifugation‐induced damage to its T cell population, it is somewhat unsurprising that the density gradient‐derived T cells did not activate nor grow as well as their CIF‐derived counterparts once put into culture. However, the 3.5‐fold advantage in overall T cell yield from the CIF approach is still notable. While there is some established evidence that microfluidic devices may damage cells to a lesser degree than centrifugation‐based methods,^31^ including our own previous studies,^13^, ^34^, ^49^ this is the first time that monocytes were essentially eliminated from the processed leukapheresis sample pre‐culture using a microfluidic device. It is possible that the combination of monocyte removal and low‐driving‐pressure/gentle flow worked synergistically to produce an isolated T cell cohort that was uniquely able to grow at a rapid rate in culture, not held back by monocyte interference/differentiation^15^, ^17^ nor accumulated cell damage from repeated pelleting by centrifugation (and the cytotoxic gradient media itself). Finally, the presence of ~4‐fold more RBCs in the CIF product may have actually contributed to faster expansion of CIF‐derived T cells in culture, as some studies have suggested a positive effect of fresh RBCs,^50^, ^51^ but not RBCs processed and stored using standard blood banking techniques,^52^ on T cell proliferation. Others have shown that a RBC:T cell ratio up to 6:1 does not significantly decrease T cell activation/proliferation,^53^ and in the case of both density gradient and CIF products, this ratio was well below 1:1.

The ability to drive the flow of sample through the CIF device with a peristaltic pump demonstrated in this study is a significant advancement because it enables processing a blood cell sample sterilely from one bag to another. Such CIF‐based systems would eliminate the need for any complex and expensive equipment, sterile cleanroom facilities, or significant human labor, and should therefore reduce the cost and complexity of the crucial cell separation steps for cellular therapy research and manufacture. CIF‐derived lymphocytes could be cultured directly (as shown here) or further enriched (e.g., via bead‐based selection for a specific cohort), and/or frozen for later use—but now with much higher purity, and concentrated into a much smaller volume. Finally, since it is a customizable/modular framework, similar CIF‐based devices may be configured to generate highly pure lymphocyte product directly from whole blood, thereby potentially eliminating the need for the costly, invasive (and itself centrifugation‐based) leukapheresis step in the future.

## AUTHOR CONTRIBUTIONS

**Anton Mukhamedshin:** Data curation (lead); formal analysis (equal); investigation (lead); project administration (supporting); validation (equal); visualization (supporting); writing – original draft (equal); writing – review and editing (supporting). **Riley C. Reddington:** Data curation (supporting); investigation (supporting); methodology (supporting); writing – original draft (supporting); writing – review and editing (supporting). **Mai T. P. Dinh:** Data curation (supporting); formal analysis (supporting); investigation (supporting); methodology (supporting); validation (supporting); visualization (supporting); writing – original draft (supporting); writing – review and editing (supporting). **Kumar Abhishek:** Data curation (supporting); formal analysis (supporting); investigation (supporting); methodology (supporting); validation (supporting); visualization (supporting); writing – original draft (supporting); writing – review and editing (supporting). **Mubasher Iqbal:** Formal analysis (supporting); investigation (supporting); visualization (equal); writing – original draft (supporting); writing – review and editing (supporting). **Marc Manheim:** Data curation (supporting); investigation (supporting); methodology (supporting); writing – original draft (supporting); writing – review and editing (supporting). **Sean C. Gifford:** Conceptualization (equal); data curation (supporting); formal analysis (equal); funding acquisition (equal); investigation (supporting); methodology (supporting); project administration (supporting); resources (supporting); supervision (supporting); validation (supporting); visualization (supporting); writing – original draft (equal); writing – review and editing (equal). **Sergey S. Shevkoplyas:** Conceptualization (equal); data curation (supporting); formal analysis (equal); funding acquisition (equal); investigation (supporting); methodology (supporting); project administration (equal); resources (equal); supervision (lead); validation (supporting); visualization (equal); writing – original draft (equal); writing – review and editing (equal).

## FUNDING INFORMATION

Research reported in this publication was supported in part by the National Institutes of Health, National Heart, Lung, and Blood Institute under Award Number R01HL151858 (Sergey S. Shevkoplyas) and the National Science Foundation, Division of Chemical, Bioengineering, Environmental, and Transport Systems under Award Number 1951153 (Sean C. Gifford). The content is solely the responsibility of the authors and does not necessarily represent the official views of the National Institutes of Health, the National Science Foundation, or the United States Government.

## CONFLICT OF INTEREST STATEMENT

Sean C. Gifford and Sergey S. Shevkoplyas are inventors of U.S. Patent #9,789,235 ‘Separation and concentration of particles’ describing the ‘controlled incremental filtration’ technology, and are co‐founders of Halcyon Biomedical, Incorporated, a company that would benefit from its commercialization. Riley C. Reddington and Marc Manheim are employees of Halcyon Biomedical, Incorporated. SSS has received research funding from Halcyon Biomedical, Incorporated. Anton Mukhamedshin, Mai T. P. Dinh, Kumar Abhishek, and Mubasher Iqbal declare no competing interests.

## Supporting information


**FIGURE S1.** COMSOL Multiphysics simulations of the fluid velocity field within a filtration gap of a CIF element. The velocity field is visualized using an arrow surface (red arrows, length normalized) and a COMSOL‐generated streamline plot (blue lines). (a) A segment of a CIF element with $f_{\mathrm{gap}}=0$, that is, a design with no net flow through the filtration gaps. Note two symmetrical recirculation zones that are formed within the gap. (b) A segment of a ‘concentrator’ element of the CIF device used in this study. Note the break in symmetry of the recirculation zones because of the non‐zero net flow through the gap (left‐to‐right). (c) A segment of a ‘separator’ element of the CIF device used in this study. Note that only one of the recirculation zones remains because of the increased flow through the gap. (d) A segment of a CIF element with $f_{\mathrm{gap}}$ higher than that for either the ‘concentrator’ or the ‘separator’ elements, and with faster flow through the gap. The recirculation zones are no longer present. See also Video S6, which shows the filtration gap examples B and C during device operation at different flow rates.
**FIGURE S2.** Assembled CIF device during operation with pulse dampeners of various volume: (a) no dampener, (b) 5 mL, (c) 10 mL, and (d) 30 mL dampener. When primed at a 5 mL/min flowrate, the trapped air within each dampener chamber is pressurized to ~5 psi with PBS buffer. When the blood sample is then introduced, relatively few cells move toward the dampener chamber with each pulse of the peristaltic pump, leaving the chamber itself as a reservoir of clarified buffer that can later be used to flush the device at the conclusion of cell processing. For this reason, a 30 mL size dampener was chosen for the experimental system investigated in this study. In microfluidic systems requiring a lesser degree of dead volume flushing, a smaller dampener size may be preferred, provided it is still capable of sufficiently attenuating the periodic pulses of the pumping mechanism used to drive flow.

**TABLE S1.** Includes raw data of cell composition, separation performance, and cell culture for each leukapheresis unit used in the study.

**VIDEO S1.** Demonstration of the operation of the CIF microfluidic system.

**VIDEO S2.** Dilute RBC suspension flowing through the microchannels of the CIF device without a pulse dampener attached to the input of the device (see Figure S2a). ‘Concentrator’ and ‘separator’ elements are shown at different magnifications operating at 5 mL/min. Note the significant outflow of retentate cells to filtrate channels during each pulse. In a representative case with no dampener, lymphocyte purity was only ~73% (compare to Video S5).

**VIDEO S3.** The same channels as shown in Video S2 but with a 5 mL pulse dampener attached to the device (see Figure S2b). Note the minor flow fluctuations still visible when using a 5 mL pulse dampener (e.g., where noted by the green arrow).

**VIDEO S4.** The same channels as shown in Video S2 but with a 10 mL pulse dampener attached to the device (see Figure S2c). Flow fluctuations when using a 10 mL pulse dampener are further reduced (e.g., where noted by the green arrow).

**VIDEO S5.** The same channels as shown in Video S2 but with a 30 mL dampener attached to the device (see Figure S2d). Flow fluctuations when using a 30 mL pulse dampener are negligible. In a representative case with a 30 mL dampener, lymphocyte purity was 89% (compare to Video S2).

**VIDEO S6.** Red cells traversing filtration gaps for both the ‘concentrator’ and ‘separator’ elements are shown for CIF devices operating at 4, 5 and 6 mL/min. No apparent change in the general filtration fluid dynamics was observed as the flowrate increased. The lymphocyte purity was ~89% for 4 and 5 mL/min, decreasing to ~87% for 6 mL/min (and further down to ~85% for 7 mL/min, and so on), indicating an outflow of an increased fraction of larger WBCs (e.g., monocytes) from the separator central/retentate flow channel to the outer/filtrate channels, due to ‘bulging’ of the outer channels caused by the increased pressure within the device.

## Data Availability

The data that supports the findings of this study are available in the supplementary material of this article.
