# Supplementary material for: Rapid, label‐free enrichment of lymphocytes in a closed system using a flow‐through microfluidic device
Source: Bioeng Transl Med. 2023 Sep 25;9(1):e10602. doi: 10.1002/btm2.10602 (PMC10771558; doi:10.1002/btm2.10602)
Supplement: Supplementary file 1 — FIGURE S1. COMSOL Multiphysics simulations of the fluid velocity field within a filtration gap of a CIF element. The velocity field is visualized using an arrow surface (red arrows, length normalized) and a COMSOL‐generated streamline plot (blue lines). (a) A segment of a CIF element with fgap=0, that is, a design with no net flow through the filtration gaps. Note two symmetrical recirculation zones that are formed within the gap. (b) A segment of a ‘concentrator’ element of the CIF device used in this study. Note the break in symmetry of the recirculation zones because of the non‐zero net flow through the gap (left‐to‐right). (c) A segment of a ‘separator’ element of the CIF device used in this study. Note that only one of the recirculation zones remains because of the increased flow through the gap. (d) A segment of a CIF element with fgap higher than that for either the ‘concentrator’ or the ‘separator’ elements, and with faster flow through the gap. The recirculation zones are no longer present. See also Video S6, which shows the filtration gap examples B and C during device operation at different flow rates. FIGURE S2. Assembled CIF device during operation with pulse dampeners of various volume: (a) no dampener, (b) 5 mL, (c) 10 mL, and (d) 30 mL dampener. When primed at a 5 mL/min flowrate, the trapped air within each dampener chamber is pressurized to ~5 psi with PBS buffer. When the blood sample is then introduced, relatively few cells move toward the dampener chamber with each pulse of the peristaltic pump, leaving the chamber itself as a reservoir of clarified buffer that can later be used to flush the device at the conclusion of cell processing. For this reason, a 30 mL size dampener was chosen for the experimental system investigated in this study. In microfluidic systems requiring a lesser degree of dead volume flushing, a smaller dampener size may be preferred, provided it is still capable of sufficiently attenuating the periodic pulses of the pumping [file BTM2-9-e10602-s005.docx]

**Supporting Information**

**Rapid, label-free enrichment of lymphocytes in a closed system using a flow-through microfluidic device**

Anton Mukhamedshin,^1^ Riley C. Reddington,^2^ Mai T. P. Dinh,^1^ Kumar Abhishek,^1^ Mubasher Iqbal,^1^ Marc Manheim,^2^ Sean C. Gifford,^2^ Sergey S. Shevkoplyas^1,*^

^1^ Department of Biomedical Engineering, University of Houston, Houston, TX 77204, USA

^2^ Halcyon Biomedical Incorporated, Friendswood, TX 77546, USA

* Corresponding author: Sergey S. Shevkoplyas, Ph.D., University of Houston, Department of Biomedical Engineering, 3605 Cullen Blvd, Houston, TX 77204-5060; phone: +1 (713) 743-5696; fax: +1 (713) 743-0226; e-mail: [sshevkoplyas@uh.edu](mailto:sshevkoplyas@uh.edu)

**Figure S1**


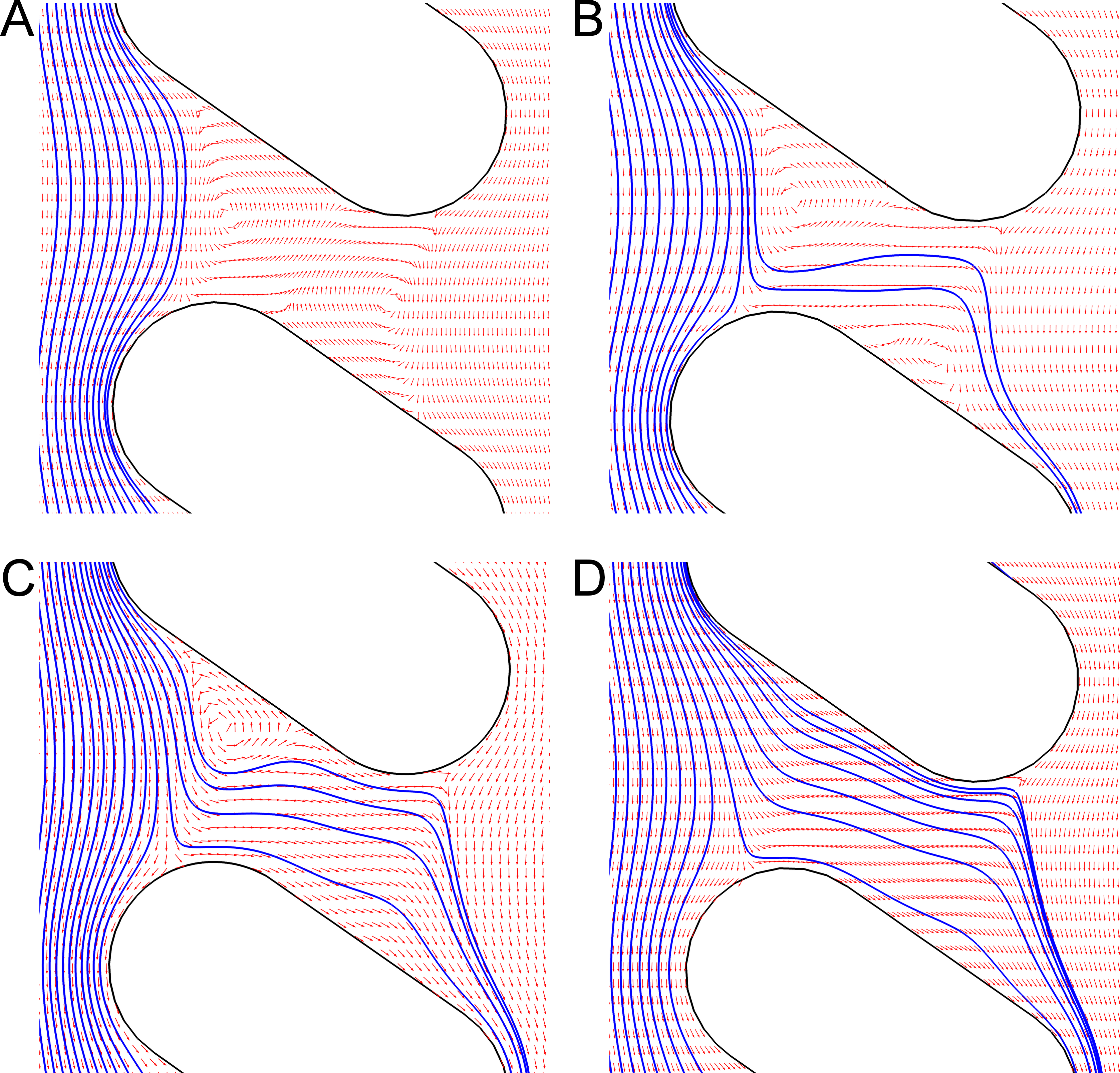


**Figure S1** COMSOL Multiphysics simulations of the fluid velocity field within a filtration gap of a CIF element. The velocity field is visualized using an arrow surface (red arrows, length normalized) and a COMSOL-generated streamline plot (blue lines). (**A**) A segment of a CIF element with $f_{gap}$ = 0, i.e. a design with no net flow through the filtration gaps. Note two symmetrical recirculation zones that are formed within the gap. (**B**) A segment of a ‘concentrator’ element of the CIF device used in this study. Note the break in symmetry of the recirculation zones because of the non-zero net flow through the gap (left-to-right). (**C**) A segment of a ‘separator’ element of the CIF device used in this study. Note that only one of the recirculation zones remains because of the increased flow through the gap. (**D**) A segment of a CIF element with $f_{gap}$ higher than that for either the ‘concentrator’ or the ‘separator’ elements, and with faster flow through the gap. The recirculation zones are no longer present. See also **Movie S6**, which shows the filtration gap examples B and C during device operation at different flow rates.

**Figure S2**


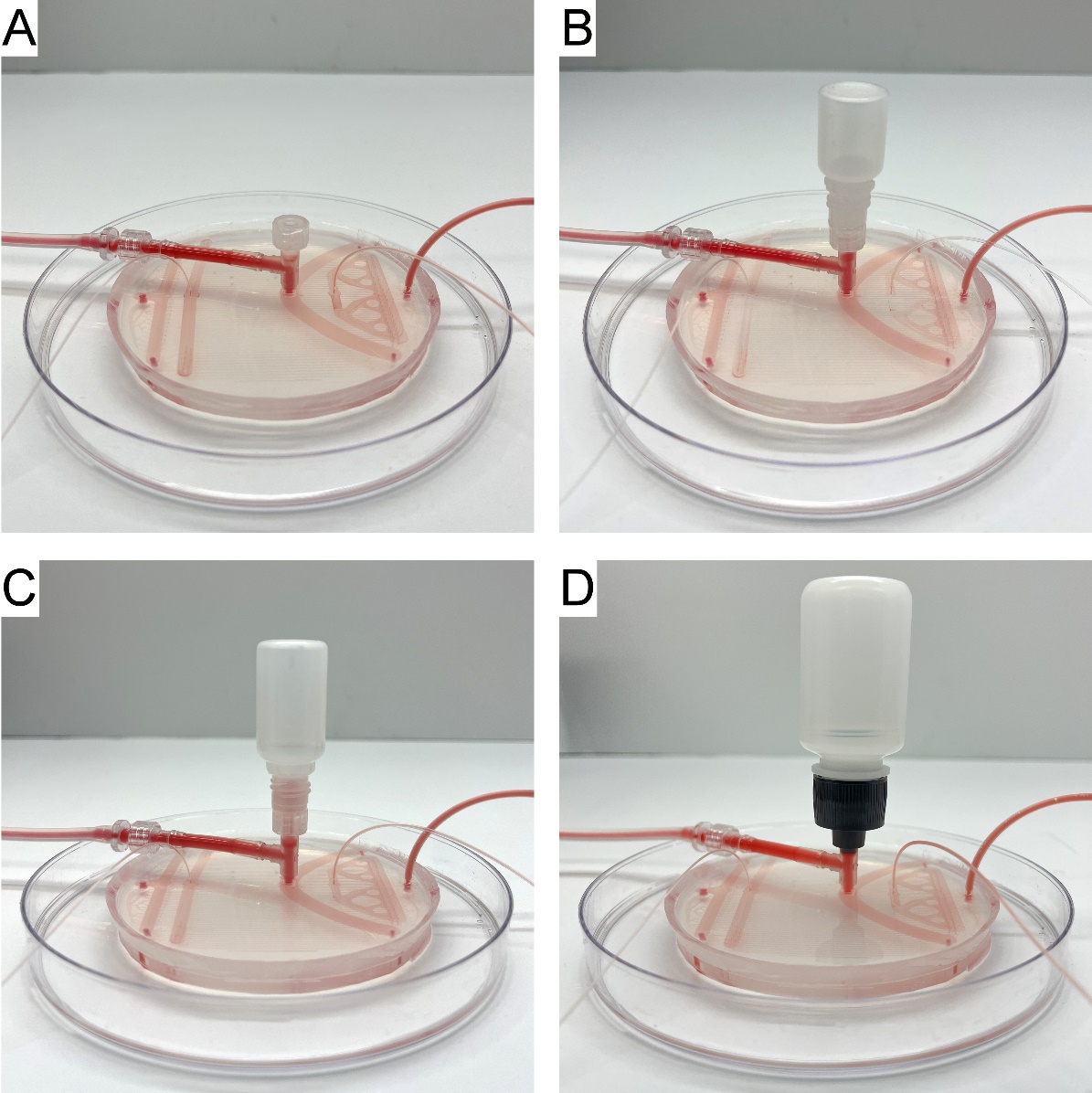


**Figure S2** Assembled CIF device during operation with pulse dampeners of various volume: (**A**) no dampener, (**B**) 5 mL, (**C**) 10 mL, and (**D**) 30 mL dampener. When primed at a 5 mL/min flowrate, the trapped air within each dampener chamber is pressurized to ~5 PSI with PBS buffer. When the blood sample is then introduced, relatively few cells move toward the dampener chamber with each pulse of the peristaltic pump, leaving the chamber itself as a reservoir of clarified buffer that can later be used to flush the device at the conclusion of cell processing. For this reason, a 30 mL size dampener was chosen for the experimental system investigated in this study. In microfluidic systems requiring a lesser degree of dead volume flushing, a smaller dampener size may be preferred, provided it is still capable of sufficiently attenuating the periodic pulses of the pumping mechanism used to drive flow.
